# Gene discovery using next-generation pyrosequencing to develop ESTs for *Phalaenopsis *orchids

**DOI:** 10.1186/1471-2164-12-360

**Published:** 2011-07-12

**Authors:** Yu-Yun Hsiao, Yun-Wen Chen, Shi-Ching Huang, Zhao-Jun Pan, Chih-Hsiung Fu, Wen-Huei Chen, Wen-Chieh Tsai, Hong-Hwa Chen

**Affiliations:** 1 Department of Life Sciences, National Cheng Kung University, Tainan 701, Taiwan; 2 Orchid Research Center, National Cheng Kung University, Tainan 701, Taiwan; 3 Institute of Tropical Plant Sciences, National Cheng Kung University, Tainan 701, Taiwan; 4 Department of Engineering Science, National Cheng Kung University, Tainan 701, Taiwan

## Abstract

**Background:**

Orchids are one of the most diversified angiosperms, but few genomic resources are available for these non-model plants. In addition to the ecological significance, *Phalaenopsis *has been considered as an economically important floriculture industry worldwide. We aimed to use massively parallel 454 pyrosequencing for a global characterization of the *Phalaenopsis *transcriptome.

**Results:**

To maximize sequence diversity, we pooled RNA from 10 samples of different tissues, various developmental stages, and biotic- or abiotic-stressed plants. We obtained 206,960 expressed sequence tags (ESTs) with an average read length of 228 bp. These reads were assembled into 8,233 contigs and 34,630 singletons. The unigenes were searched against the NCBI non-redundant (NR) protein database. Based on sequence similarity with known proteins, these analyses identified 22,234 different genes (E-value cutoff, e^-7^). Assembled sequences were annotated with Gene Ontology, Gene Family and Kyoto Encyclopedia of Genes and Genomes (KEGG) pathways. Among these annotations, over 780 unigenes encoding putative transcription factors were identified.

**Conclusion:**

Pyrosequencing was effective in identifying a large set of unigenes from *Phalaenopsis*. The informative EST dataset we developed constitutes a much-needed resource for discovery of genes involved in various biological processes in *Phalaenopsis *and other orchid species. These transcribed sequences will narrow the gap between study of model organisms with many genomic resources and species that are important for ecological and evolutionary studies.

## Background

The family of Orchidaceae is the largest family of flowering plants and the number of species may exceed 25,000 [1]. Like all other living organisms, present-day orchids have evolved from ancestral forms as a result of selection pressure and adaptation. They show a wide diversity of epiphytic and terrestrial growth forms and have successfully colonized almost every habitat on earth. Factors promoting orchid species richness include specific interaction between the orchid flower and pollinator [2], sequential and rapid interplay between drift and natural selection [3], obligate interaction with mycorrhiza [4], and epiphytism which is true for most of all orchids and probably two-thirds of the epiphytic flora of the world.

The radiation of the orchid family has probably taken place in a comparatively short period as compared with that of most flowering plant families, which had already started to diversify in the Mid-Cretaceous [5]. The time of origin of orchids is in dispute, although Dressler suggests that they originated 80 to 40 million years ago (Mya; late Cretaceous to late Eocene) [6]. Recently, the origin of the Orchidaceae was dated with a fossil orchid and its pollinator. The authors showed that the most recent common ancestor of extant orchids lived in the late Cretaceous (76-84 Mya) [7]. They also suggested that Epidendroideae and Orchidoideae, two of the largest orchid subfamilies, which together represent > 95% of living orchid species, began to diversify early in the Tertiary (65 Mya) [7].

According to molecular phylogenetic studies, Orchidaceae comprise 5 subfamilies: Apostasioideae, Cypripedioideae, Vanilloideae, Orchidoideae and Epidendroideae. The Apostasioideae is considered the sister group to other orchids. Vanilloideae diverged just before Cypripedioideae. Both subfamilies have relatively low numbers of genera and species. Most of the taxonomic diversity in orchids is in 2 recently expanded sister-subfamilies: Orchidoideae and especially Epidendroideae [8,9]. Orchids are known for their diversity of specialized reproductive and ecological strategies. For successful reproduction, the production of labellum and gynostemium (a fused structure of androecium and gynoecium) to facilitate pollination is well documented and the co-evolution of orchid flowers and pollinators is well known [10,11]. In addition, the especially successful evolutionary progress of orchids may be explained by mature pollen grains packaged as pollinia, pollination-regulated ovary/ovule development, synchronized timing of micro- and mega-gametogenesis for effective fertilization, and the release of thousands or millions of immature embryos (seeds without endosperm) in a mature capsule [12]. However, despite their unique developmental reproductive biology, as well as specialized pollination and ecological strategies, orchids remain under-represented in molecular studies relative to other species-rich plant families [13]. The reasons may be associated with the large genome size, long life cycle, and inefficient transformation system of orchids.

The genomic sequence resources currently available for orchids are limited. Very recently, a sketch of the *Phalaenopsis *orchid genome from sequencing the ends of 2 bacterial artificial chromosome libraries of *P. equestris *was reported [14]. In addition, a number of studies have developed expressed sequence tags (ESTs) resources for orchids by using Sanger sequencing [15-18]. Fewer than 12,000 ESTs, including 5,593 from *P. equestris*, 2,359 from *P. bellina*, 1,080 from *Oncidium *Gower Ramsey, and 2,132 from *Vanda *Mimi Palmer, have been deposited in public database. These studies have highlighted the utility of cDNA sequencing for discovering candidate genes for orchid floral development [19,20], floral scent production [16,21] or flowering time [22] in the absence of a genomic sequence. However, a comprehensive description of the full complement of gene expressed in orchids remains unavailable.

Massively parallel 454 pyrosequencing has become feasible for increasing sequencing depth and coverage while reducing time, labour, and cost [23,24]. This technology can be used to deeply explore the nature and complexity of a given transcriptional universe. 454 sequencing of transcriptomes for model organisms has confirmed that the relatively short reads produced by this technology can be effectively assembled and used for gene discovery [25,26]. In addition, the superior performance of this technology has been demonstrated in several studies [27], including those of mustard weed *Arabidopsis thaliana *[28,29], the model legume *Medicago truncatula *[21], maize *Zea may *[30,31], the tree *Eucalyptus grandis *[32], chestnut [33], oil crop *Olea europaea *[34], oilseed rape *Brassica napus *[35], and the antimalarial plant *Artemisia annua *[36].

The genus *Phalaenopsis *Blume (Orchidaceae), a beautiful and one of the most popular ornamental flowers exported worldwide, comprises 66 species according to the latest classification by Christenson [37]. The species are found throughout tropical Asia and the larger islands of the Pacific Ocean. In Taiwan, 2 of the native species, *P. equestris *and *P. aphrodite *subsp. *formosana*, are usually used as parents for breeding. *P. equestris *possesses several favorable commercial traits such as numerous spikes and branches and multitudinous and colorful flowers. *P. aphrodite *subsp*. formosana *has a perfect arrangement of flower positions at the spike and an elegant flower shape with extended longevity. The flowers of both species are scentless. Many of the scent traits in the *P*. hybrids are mainly derived from *P. bellina *and/or *P. violaceae*, the native species in Malaysia. Both *P. equestris *and *P. aphrodite *subsp*. formosana *are diploid plants with 38 chromosomes (2n = 2x), which are small and uniform in size (< 2 μm). The estimated haploid genome sizes are 1,600 Mb (3.37 pg/diploid genome) and 1,300 Mb (2.80 pg/diploid genome) for *P. equestris *and *P. aphrodite *subsp*. formosana*, respectively, which are relatively small in genus of *Phalaenopsis *[38]. The 2 species could be considered model organisms for studying orchid biology because of their relative small genome size [39], high performance of culture system and well applicable functional genomic tools such as genetic transformation [40-42] and virus-induced gene silencing system [43].

In this report, we provide the first comprehensive characterization of the transcriptome of *Phalaenopsis *orchids by massively parallel 454 pyrosequencing. To expansively cover the *Phalaenopsis *orchid transcriptome and facilitate identifying sets of genes involved in a broad range of biological processes, we developed the EST set from 10 samples derived from 3 different species of *Phalaenopisis *(Table 1). Based on data corresponding to a single run on the GS FLX sequencer, almost 42 million bases were assembled into ~43,000 pieces of putative transcripts and the majority of these have been annotated and functionally classified. All the sequences were deposited in OrchidBase (http://140.116.25.218/EST, [44]) and Short Read Archive (SRA) division of the GenBank repository (accession no. SRA030758.2). These results can provide the means for future genome-wide orchid biology and biotechnology research in *Phalaenopsis*.

**Table 1:** Samples used for transcriptome analysis

Species	Tissues
*Phalaenopsis equestris*	inflorescence
	flower bud
	root
	young leaf
	old leaf
	cold stressed leaf
	*Erwinia chrysanthemi *-infected leaf
*Phalaenopsis aphrodite*	protocorm
	cold night temperature -induced spike
*Phalaenopsis bellina*	Day 5 post anthesis flower

## Results

### Sequencing and assembly of 454 pyrosequenced ESTs

**Figure 1:**
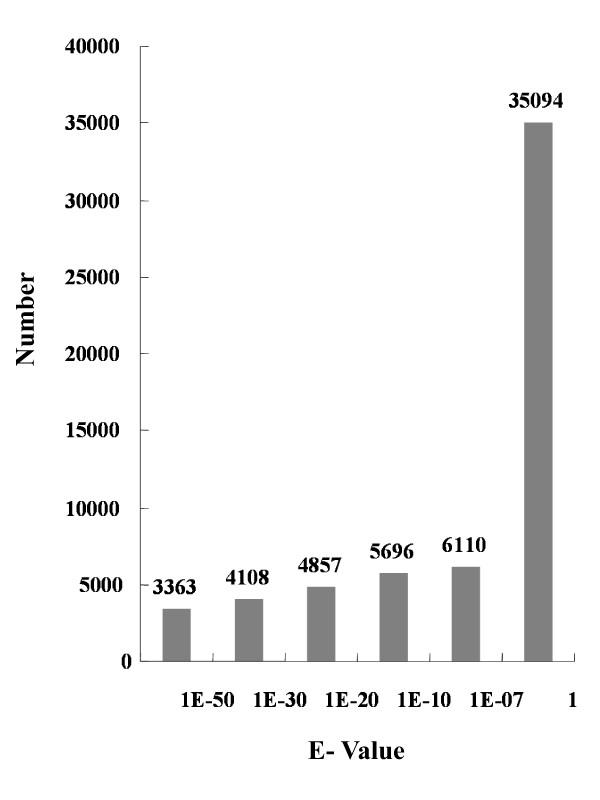
**Comparison of sequences generated by use of 454 sequencing and *Phalaenopsis *ESTs published in the NCBI EST database by BLASTN**.

**Figure 2:**
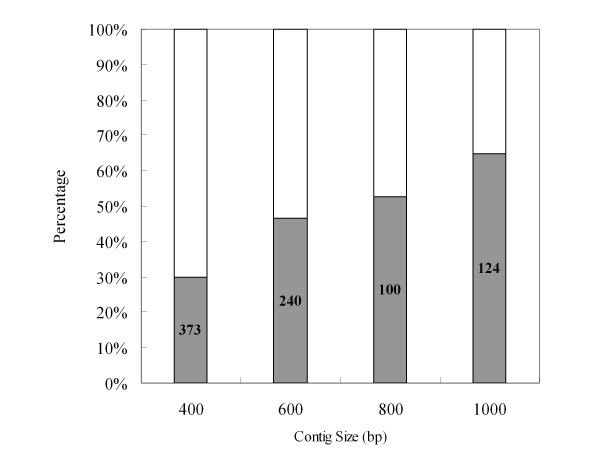
**Comparison of percentage of hits among different sized contigs against *Phalaenopsis *ESTs published in the NCBI EST database by BLASTN (E-value < 10^-10^)**.

In total, 207,110 ESTs (minimal size > 50 bp) averaging 230 bp were generated from one pyrosequencing run. Cleaning (removal of primer, polyA tail, etc) of the raw sequences resulted in a total of 206,960 high-quality reads with an average length of 228 bp nucleotides totaling 42 Mb. After assembly using GS FLX gsAssembler, these reads were assembled into 8,233 contigs and 34,630 singletons under the criteria of minimum 40 bases overlap with at least 95% identity. The average length of contigs and singletons was 364 bp and 201 bp, respectively. These contigs and singletons represented up to 42,863 unigenes. The length distribution of unigenes and their component reads are summarized in Table 2 and Additional file 1 and Additional file 2. Our assembly also included a larger proportion of long contigs (16% of the contigs were ≥ 500 bp) (Additional file 1) than the 8% reported by Novaes et al. [32]. The unigene set was also compared to the ESTs derived from *P. equestris *[11] and *P. bellina *[12]. About 16.2% (E-value cutoff, e^-10^) of the unigenes could be matched to the published *Phalaenopsis *ESTs (Figure 1). In addition, different sized contigs were selected to recalculate the BLAST hits. Result showed that the percentage of hits was proportion to contig size (400 bp-600 bp, 29.84%; 600 bp-800 bp, 46.6%; 800 bp-1000 bp, 52.63%; > 1000 bp, 64.92%; Figure 2). Furthermore, the previously available ESTs derived from *Phalaenopsis *orchids were BLAST against ESTs generated by this study. Results showed that among 4,875 unigenes, 3,503 (71.9%) matched the ESTs produced by 454 pyrosequencing.

**Table 2:** Summary of *Phalaenopsis *EST data

Total Bases	42,034,787
High-quality Reads	206,960
Average Read Length	228
Number of Contigs	8,233
Average Contig Length	364
Range of Contig Length	72 to 4234
Number of Reads in Contigs	172,330
Number og Singletons	34,630
Number of Unigene Sequences	42,863

**Figure 3:**
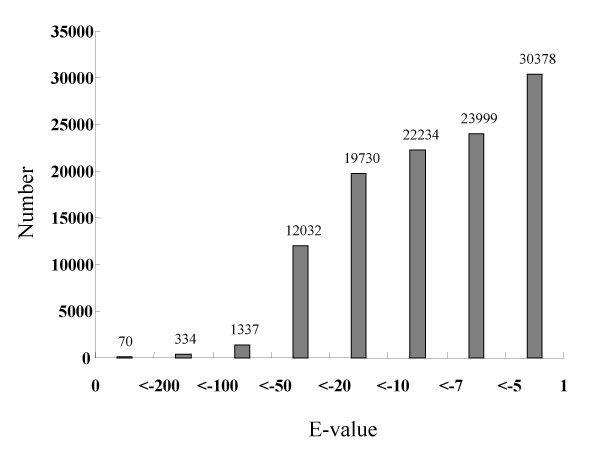
**The number of sequence hits against the NCBI non-redundant (NR) database by BLASTX**.

**Figure 4:**
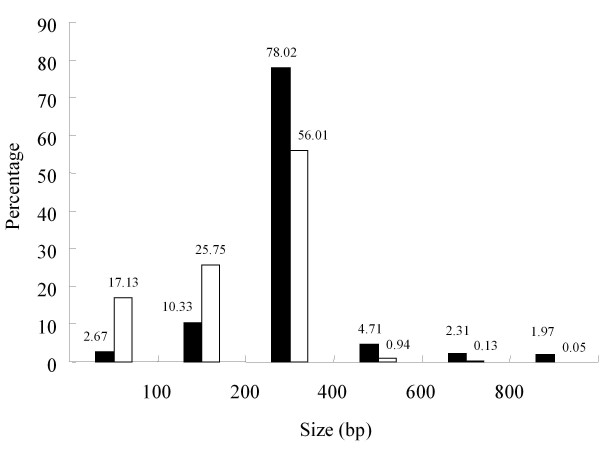
**Comparison of percentage of hits in the NR database for different sized ESTs with (black bars) and without (white bars) homology (E-value < 10^-7^)**.

The unigenes were searched against the NCBI non-redundant (NR) protein database by use of the BLASTX algorithm. Among the 42,863 unigenes, 22,234 (51.9%) had at least one significant alignment to existing genes in the database (E-value cutoff, e^-7^) (Figure 3), and 20,684 of these (93.03%) had an open reading frame (ORF). A majority (48.1%) of the pyrosequencing assemblies did not match any known sequences in the database. To analyze effects of fragment size on annotation efficiency, we compared the percentage of hits between different sized ESTs with and without homology at E-value < 10^-7 ^(Figure 4). The result showed that 42.88% of genes without homology and 13% of those with homology were < 200 bp. Thus, short reads may have a low level of annotation. In addition, it is also possible that unannotated reads may likely represent novel genes. Alternatively, these sequences might correspond to divergent 5' or 3' regions of genes.

### Functional annotation of novel transcripts

**Figure 5:**
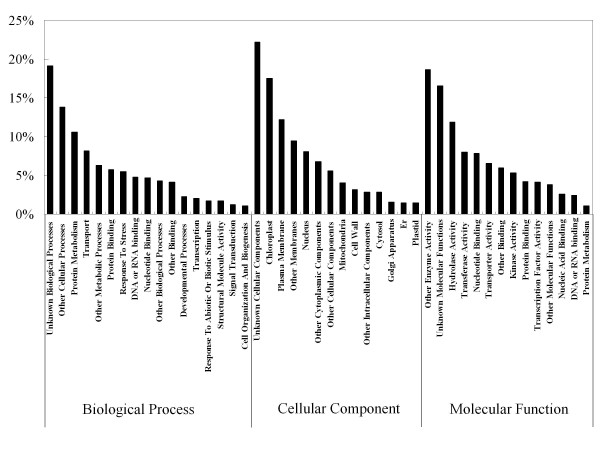
**Representation of genome ontology assignments for *Phalaenopsis *ESTs derived from 454 sequencing**. The GO Slim Classification for Plants developed at TAIR was used to characterize the ESTs functionally.

To determine the possible functions of genes tagged, we used the Gene Ontology (GO) classification system for plants developed at TAIR [45] (http://www.arabidopsis.org/help/helppages/go_slim_help.jsp). The functions of genes identified cover various biological processes, molecular functions and cellular components (Figure 5). In total, 10,427 of the 42,863 transcripts were assigned to biological processes. The largest proportion of functionally assigned ESTs fell into protein metabolism (11%) and transport (8%) except of unknown biological processes (20%) and other cellular processes (14%). Among the molecular functions, 9,518 out of a total 42,863 transcripts were assigned in this category. The molecular functions most represented were hydrolase activity (12%), transferase activity (8%) and nucleotide binding (8%) except for other enzyme activity (19%) and unknown molecular functions (17%). Among the cellular components, 9,685 out of a total 42,863 transcripts were assigned in this category. Chloroplast (18%) and plasma membrane (12%) are the most represented categories in cellular components except for unknown cellular components (22%).

These ESTs obtained from a non-normalized cDNA library could be used to reveal global gene expression patterns as deduced from transcript abundance. The transcripts highly expressed in *Phalaenopsis *tissues are listed in Table 3. The most abundant ESTs coded for cytochrome P450-like protein precursor (2.27%), followed by triple gene block 3 (1.98%) and cytochrome P450 monooxygenase (1.28%). These ESTs probably correspond to the most highly expressed genes in the *Phalaenopsis*. Plant systems utilize a diverse array of cytochrome P450 monooxygenases in their biosynthetic and detoxicative pathways. High expression of the cytochrome P450 gene family suggests that these genes are important for orchid subsistence and adaptation to the environment. The high expression of triple gene block 3 raised the interesting question of whether the sequence samples were infected by *Cymbidium *mosaic virus (CymMV) or if the gene is included in the *Phalaenopsis *genome. Therefore, we screened other genes of the same CymMV in the EST dataset. In addition to triple gene block 3, several genes of CymMV were found, including 30 unigenes of RNA dependent RNA polymerase, 24 of coat protein, 4 of triple gene block 1, and 2 of triple gene block 2. These transcripts showed significant expression in *Phalaenopsis*, which indicates that some of the experimental materials used here may have been infected with virus prior to the sampling.

**Table 3:** Highly abundant transcripts detected in *Phalaenopsis*

Putative Function	Organism	E-value	Number of Component Reads	Number of Contigs (97% identity)	Number of Contigs (99% identity)
Putative P450- like protein precursor	Zea mays	5.00E-20	4,705	2	4
Triple gene block 3	Cymbidium mosaic virus	3.00E-22	4,100	n.d.	n.d.
Cytochrome P450 monooxygenase	Sorghum bicolor	2.00E-08	2,653	5	7
LLA- 1378	Lilium longiflorum	3.00E-28	2,269	3	3
Hypothetical protein	Sorghum bicolor	3.00E-15	1,668	3	4

n.d.: not determined

Analysis of a large number of ESTs has revealed ancient polyploidy throughout the major angiosperm lineages [46,47]. It would be interesting to analyze how many subfamilies exist in very high number of ESTs to evaluate the possibility of gene duplication in *Phalaenopsis *orchids. We set more stringent criteria for assembly (a minimum of 40 bases of overlap with 97% and 99% identity) and found a greater number of unigenes (Table 3), suggesting that some genes may have been undergone gene duplication. However, more evidence is needed to solve the causes that lead to the formation of paralogous genes, such as whole-genome duplication, tandem gene duplication or segmental duplication.

### Gene families and Pathways

To evaluate the effectiveness of the orchid transcriptome library, we categorized the assembled unigenes through the use of *Arabidopsis *proteome as targets. In total, 4,833 unigenes were classified into 130 gene families representing 73.9% (130/176) of *Arabidopsis *gene families (Additional file 3). We also mapped assembled unigenes into the KEGG Pathways including metabolism, genetic information processing, environmental information processing, cellular processes, and organism systems (http://www.genome.jp/kegg/pathway.html). A total of 7,885 unigenes were mapped onto KEGG Pathways (Table 4). Among these, 6,269 unigenes were related to metabolism, 1,078 unigenes corresponded to genetic information processing, 121 unigenes mapped to environmental information processing, 213 unigenes were classified as cellular processes, and 204 unigenes belonged to organism systems (Table 4). The presence genes for these essential cellular processes suggest that these sequences account for most of the comprehensive *Phalaenopsis *transcriptome.

**Table 4:** Unigenes mapped in KEGG Pathways

KEGG Pathways	Sub-pathways of KEGG Pathway	Number of Unigenes	Number of reads
Metabolism		6269	43325
	Glycan Biosynthesis and Metabolism	84	137
	Xenobiotics Biodegradation and Metabolism	22	95
	Metabolism of Other Amino Acids	162	675
	Biosynthesis of Polyketides and Terpenoids	146	596
	Carbohydrate Metabolism	1229	7957
	Overview	2360	19261
	Biosynthesis of Other Secondary Metabolites	179	1201
	Lipid Metabolism	450	1860
	Nucleotide Metabolism	236	678
	Metabolism of Cofactors and Vitamins	188	707
	Amino Acid Metabolism	639	2998
	Energy Metabolism	574	7160
Genetic Information Processing		1078	6944
	Replication and Repair	149	384
	Transcription	301	1962
	Folding, Sorting and Degradation	329	1674
	Translation	299	2951
Organismal Systems		204	828
Cellular Processes		213	2167
Environmental Information Processing		121	460

**Figure 6:**
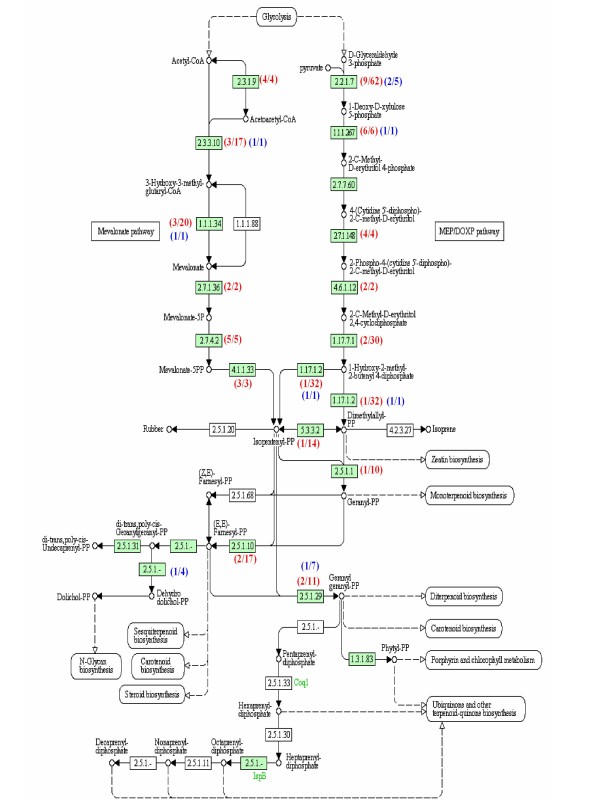
**Graphic representation of terpenoid backbone biosynthesis pathways, including cytosolic mevolonate pathway and plastic methylerythritol phosphate pathway, for *Phalaenopsis *orchids**. The red and blue numbers represent the ESTs generated by this study and derived from *P. bellina *[16], respectively, that are homologous to genes involved in the terpenoid backbone biosynthesis pathway. The first number in the bracket is the number of unigenes corresponding to the catalytic gene in the pathway, and the second number is the number of reads for the unigene.

Previously, we identified the major compounds emitted from *P. bellina *flowers to be monoterpenes, including linalool and geraniol [13]. Identification of the potential genes involved in the biosynthetic pathways for terpenoid precursors will be important for understanding the regulation of scent biosynthesis in orchids. In our 454 sequence dataset, 50 unigenes assembled from 209 reads were found potentially related to the methylerythritol phosphate pathway (MEP) and mevalonate pathway (MVA) (Additional file 4). Analysis of previous ESTs from *P. bellina *showed 8 unigenes assembled from 20 reads were potentially related to the MEP and MVA pathways (Figure 6). The use of 454 pyrosequencing identified more genes and more ESTs per gene constituted in the terpenoid backbone biosynthesis than did Sanger sequencing (Figure 6). At least one unigene was found to correspond to the enzymes participating in the two pathways, except the third step of the MEP pathway (Figure 6). Thus, the large amount of transcriptomic information provided by 454 pyrosequencing may accelerate the progress for the study of scent production and regulation in orchids.

### Transcription factors

Because transcription factors control the expression of a genome and play important roles in all aspects of the life cycle of higher plants, we characterized the transcription factor-associated ESTs from the transcriptome of *Phalaenopsis *by using rice transcription factor sequences downloaded from the Plant Transcription Factor Database (http://planttfdb.cbi.pku.edu.cn/) as queries. In the *Phalaenopsis *transcriptome, we identified 786 unigenes consisted of 2,317 reads encoding putative transcription factors, occupying 1.83% (786/42,863) of the unigenes of *Phalaenopsis *transcriptome. Compared to the 5.7% of plant genes that have been shown to be transcription factor genes [48], 1.83% of genes related to transcription factors in *Phalaenopsis *is low. To analyze the underestimation, we mapped all unigenes to 9 full-length cDNAs (FL-cDNAs) encoding MADS-box proteins (PeMADS1~PeMADS9) derived from *P. equestris*. In total, 33 unigenes could be mapped to the 9 FL-cDNAs. Among the 33 unigenes, 5 are located in untranslated regions, and the other two are short fragments (< 90 bp) located within coding regions. However, these seven unigenes were not identified as transcription factor genes. These results suggest that one of the reasons for an underestimation of transcription factor genes in *Phalaenopsis *can be explained by sequences corresponding to divergent 5' or 3' regions of genes and/or they are short reads *per se*.

**Figure 7:**
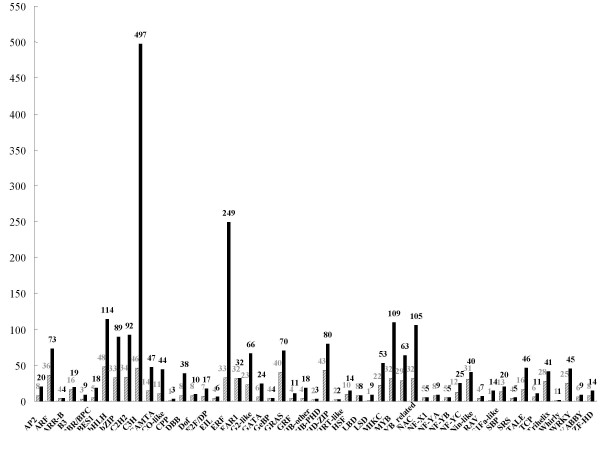
**Number of ESTs related to transcription factors in each transcription factor family**. A total of 2,424 putative *Oryza sativa *subsp*. japonica *transcription factors were searched against the *Phalaenopsis *transcriptome, and target ESTs were classified into corresponding transcription factor families. Black bars are the number of reads in each family. Shaded bars are the number of unigenes in each family.

The most abundantly expressed transcription factor gene families, C3H and AP2/ERF, accounted for a full 32.2% (746/2,317) of the overall transcription factor expression (Figure 7). In addition, bHLH (4.9%), MYB (4.7%) and NAC (4.5%) families were found in turn (Figure 7). These five families occupied approximately 46.3% of expressed transcription factors. However, five families of transcription factors including LFY, M-type, STAT, VOZ, and WOX were not detected. The few families that could not be found might result from inadequate sampling of the transcriptome or genes that are truly rarely expressed. Large-scale efforts in deeper sampling and sequencing of the transcriptome will help completely identify genes related to of transcription factor families in orchids.

## Discussion

As the most species rich and diversified family, Orchidaceae has mesmerized botanists for centuries. As for most other non-model plant species, we lack genetic and genomic resources for molecular biological study. Although a precise estimate of transcriptome coverage is unattainable without the full genomic sequence, the massively parallel pyrosequencing characterization can be considered an initial step for functional genomics studies in *Phalaenopisis*. From 206,960 sequence reads, we assembled data for 42,863 unigenes consisting of 8,233 contigs and 34,630 singletons from *Phalaenopsis*. Although a high number of transcripts are short-length reads which may result in several assembled contigs and singletons for each gene, the dataset we report here still provides a plentiful dataset of different genes representing a substantial part of the transcriptome of orchids, which in turn reflects these plants' sophisticated designs for successful pollination, reproduction and adaptation to the environment.

Homology searches showed that 48.1% of the ESTs have no significant similarities to any other protein sequences in public databases. About 42.88% of these ESTs are < 200 bp, indicating that the short size has a negative effect on successful annotation. However, these genes may perform specific roles in orchids and be quite divergent from those of other plant species. The orchids, indeed, have diverse specialized reproductive and ecological strategies for adaptive radiation. On the other hand, we could not reliably annotate a high proportion of unigenes lacking assignment of a putative function because they did not cover the full length of the transcript or because they represent untranslated regions.

Comparing the distributions of the functional categories among ESTs provides support for the expression levels of the different gene classes. The transcripts with the first and third highest expression we found for *Phalaenopsis *were homologous to the members of cytochrome P450 (4.55%). Plant cytochromes P450 catalyze a wide variety of monooxygenation/hydroxylation reactions in primary and secondary metabolism. Genomic sequencing projects have revealed that cytochromes P450 genes represent approximately 1% of the total gene annotations for each plant species [49,50]. In addition to revealing the highest transcript expression in *Phalaenopsis*, the next-generation transcriptome sequencing also generated 94 members of the cytochrome P450 family. The extraordinary expression level and remarkable diversification of this gene family may have led to the *Phalaenopsis *orchid survival ability. The transcript with the second highest level of expression was homologous to triple gene block 3 (1.98%) of CymMV. We also found genes with significant expression that were homologous to RNA dependent RNA polymerase, coat protein, triple gene block 1 and triple gene block 2 of CymMV. Even though we found no substantial virus-infected symptoms in our samples, some of the experimental materials have been infected with virus prior to the sampling. We also found transcripts with high expression (1.1%) that were homologous to LLA-1378 derived from lily (*Lilium longiflorum*). This transcript is found in immature anther, tepal, pistil, stem and leaf in lily [51], however it has an as yet unknown function. Dissection of function of these genes might be a useful direction for further study of orchid biology.

The fact that whole-genome duplication often gives rise to species-rich groups of organisms, such as > 23,000 species of Asteraceae and > 19, 400 species of Fabaceae, highlights that polyploidy can facilitate diversification and speciation of organisms [52,53]. The Orchidaceae contains more than 25,000 species and has successfully colonized almost every habitat on earth. Whole-genome duplication may also have occurred in the orchid genome. Based on the results from analyzing how many subfamilies exist with very high number of ESTs, we suggested gene duplication probably have occurred on these genes. However, these gene duplication events may be caused by whole-genome duplication, tandem gene duplication or segmental duplication. Only after completeness of whole-genome sequencing of *Phalaenopsis *has been performed, it will be possible to differentiate whole-genome duplications from segmental and tandem duplications by mapping chromosome locations of the duplicated genes or blocks of genes.

To evaluate whether the sequences annotated in this study include all genes expressed in these tissues, developmental stages, and treatments, we searched for a number of genes involved in metabolic pathways and homologous to members characterized in *Arabidopsis *gene families. The genes associated with metabolic pathways were based on the KEGG database of pathways and those for gene family on the TAIR database of *Arabidopsis *proteome. The rationale for these searches was that those essential genes must be expressed to maintain cellular functions, so failure to find these sequences in the transcriptome would reflect either inadequate sequencing depth or ineffective annotation. For the pathways considered here, essentially all genes involved in the pathways were found except those involved in anthocyanin biosynthesis. For the 176 gene families in Arabidopsis, total 4,833 unigenes were classified into 130 gene families occupying more than 70% (130/176) of *Arabidopsis *gene families. A caution for these finding is that high levels of expression might be expected for some essential house-keeping genes, leading them to be well represented in even an incomplete transcriptome sequencing effort. To account for this possibility, we searched for genes associated with transcription factors. Because of their more restricted spatial and temporal expression profiles, transcription factor genes are not expected to be as highly expressed as essential house-keeping genes in whole-organism libraries. We successfully identified genes from nearly all the transcription factor families considered (51/56, 91%). The few genes that could not be found might result from incomplete annotation or inadequate sampling of the transcriptome or they may be truly not expressed. Overall, these searches support that the collection of annotated sequences we produced represents a reasonably broad description of the *Phalaenopsis *transcriptome. These sequences of expressed transcripts will be very useful for genome annotation of *Phalaenopsis *genome in the future.

Analysis of expression profiles of transcription factors in the transcriptome of *Phalaenopsis *is meaningful because these are master-control proteins in all living cells. Surprisingly, we found that C3H and AP2/ERF families together represented more than 30% of expression of *Phalaenopsis *transcription factors. The C3H family has been reported to be involved in *Arabidopsis *embryogenesis [54], shoot apical meristem maintenance [55], drought tolerance [56], and response to abscisic acid in *Craterostigma plantagineum *[57]. The AP2/ERF superfamily is defined by the AP2/ERF domain, of about 60 to 70 amino acids, and is involved in DNA binding. A combination of genetic and molecular approaches has been used to characterize a series of regulatory genes of the AP2/ERF family. The members of this family are involved in regulating various biological processes related to growth and development, as well as various responses to environmental stimuli. This family includes genes related to drought [58], high salt concentration [58], low temperature [59], diseases [60,61], and the control of ovule development and flower organ growth [62]. Understanding the functions of these genes will advance our understanding of the great morphological diversity and successful adaptation of orchids. However, we did not find the transcription factor families LFY, M-type, STAT, VOZ, and WOX, in the *Phalaenopsis *transcriptome. These families might either be rarely expressed, or might not have appeared in our cDNA sampling.

## Conclusion

Thanks to recent advances in next-generation sequencing technology, we have applied RNA-seq to facilitate transcriptome analysis of orchids which present important biological questions but lack a fully sequenced genome. Our findings represent substantial contributions to the publicly accessible expressed sequences for the Orchidaceae family. With the whole genome sequencing of *P. equestris *in progress, this collection of ESTs is a valuable resource that will be immediately useful for researchers, allowing for correction of assemblies, annotation, and construction of gene models to establish accurate exon-intron boundaries. Application of these resources through the common language of nucleotide sequences will greatly enhance the insights into the reproductive success of orchids.

## methods

### Plant materials and cDNA library construction

*Phalaenopsis equestris*, *P. aphrodite *subsp*. formosana *and *P. bellina *were grown without fungal symbiosis in greenhouses at National Cheng Kung University under natural light and controlled temperature ranging from 23°C to 27°C. To maximize the diversity and effectively collect sequences from expressed genes of orchids, we collected 10 samples from different tissues, developmental stages and treatments (Table 1). Inflorescences, flower buds, leaves and roots were sampled from the 3-year-old *P. equestris*. Young leaves were collected as they emerged. Old leaves were taken at the fourth leaf counting down from the newly emerged one. The cold-stressed leaves were collected from old leaves of 3-year-old plants treated for 4 hrs at 4°C. Because *Erwinia chrysanthemi *is one of the most serious pathogens infecting *Phalaenopsis*, old leaves were infected with *E. chrysanthemi *to induce the expression of pathogen-related genes. Protocorms were 20-day-old germinating seeds of *P. aphrodite *subsp*. formosana *grown on tissue-cultured plates without fungal symbiosis. Cool night-induced spikes were sampled from 3-year-old *P. aphrodite *subsp*. formosana *treated with cool night temperature (28°C day/20°C night) for 2 weeks to induce spike emergence [63]. *P. bellina *flowers with a strong fragrance were collected on day 5 post-anthesis [16]. Collected samples were frozen immediately in liquid nitrogen and stored at -80°C until used.

Total RNA from each sample was extracted separately following the method described by [19]. Poly-A RNA was prepared from each total RNA sample using the Oligotex^@ ^mRNA Mini kit (Qiagen, Ontario, Canada). Samples of 0.5 μg mRNA from each sample were combined into a single large pool and mixed well. This single large, equally-mixed pool was the source for the cDNA library construction. The cDNA library was constructed using the SMART cDNA synthesis Kit (BD Clontech, Mountain View, CA) according to the manufacturer's instructions.

### Pyrosequencing and assembly

In preparation for 454 sequencing, 5 μg of the cDNA sample was nebulized to a mean fragment size of 600 ± 50 bp, end repaired and adapter ligated according to previously published literature [23]. After streptavidin bead enrichment and DNA denaturation, single-stranded molecules were titrated onto derivatized Sepharose beads and then amplified by emulsion PCR. A second streptavidin bead enrichment followed emulsion breaking, the bead-attached DNAs were then denatured with NaOH, and sequencing primers were annealed. One 454 pyrosequencing run was carried out with use of a GS FLX sequencer. A 454 SFF file containing raw sequences and sequence quality information can be access through the SRA web site under accession number SRA030758.2.

Low quality data (base call score < 10) were trimmed from the ends of individual sequences. Sequences shorter than 50 bp after processing were excluded from the analysis. For assembly, GS FLX gsAssembler was used with minimum 40 bases overlap with at least 95% identity.

### Sequence analysis and GO classification

All sequences were queried for their similarity to known sequences by use of a BLASTX algorithm [64] against the "nr" protein database. Sequence similarity was considered significant at E-value < 10^-7 ^and the "best hits" annotation was used to represent proteins similar to those encoded by the contigs and singletons. The BLAST score (bits) used the BLOSUM 62 matrix and Existence 11, Extension 1 Gap costs for BLASTX. The GO Slim Classification for Plants, developed at TAIR (http://www.arabidopsis.org/help/helppages/go_slim_help.jsp) was used to characterize the ESTs functionally. The GO identifier of the best hit (with a cutoff of 1e-7) was attributed to the sequence. This step allowed putative functions to be assigned on the basis of the classification proposed by GO.

### Characterization of ESTs by Arabidopsis Gene Family and KEGG Pathways

The TAIR9 *A. thaliana *annotated protein databases (ftp://ftp.arabidopsis.org/home/tair/Genes/TAIR9_genome_release/TAIR9_sequences) was downloaded. The protein sequence set was BLAST against *Phalaenopsis *contigs and singletons with use of the TBLASTN programs. Sequence similarity was considered significant at an E-value < 10^-7^. Unique sequences with BLAST matches were mapped to TAIR Gene Families and KEGG Pathways of *Arabidopsis *for further analysis. The TAIR Gene Family information contains 8,693 genes in 176 gene families updated on September 26, 2009. The KEGG Pathways for *Arabidopsis *contains 6,756 genes in 121 pathways released on May 11, 2010.

### Identification of putative transcription factor-related ESTs

The protein sequences of predicted transcription factors for rice were downloaded from the Plant Transcription Factor Database (PTFDB; http://planttfdb.cbi.pku.edu.cn/). PTFDB contains information on 2,424 rice (*Oryza sativa *subsp*. japonica*) transcription factors in 56 families. For identification of transcription factor-related ESTs from *Phalaenopsis*, the protein sequence set of each predicted rice transcription factor family was BLAST against *Phalaenopsis *contigs and singletons with use of the TBLASTN programs. Sequence similarity was considered significant at E-value < 10^-7^.

## Authors' contributions

YYH conceived the study and design, participated in the library construction and data analysis. YWC performed the design of the bioinformatic analyses. SCH carried out RNA extraction and cDNA synthesis. ZJP contributed to the sample collection. CHF constructed the platform for displaying metabolic pathway. WHC suggested and offered the orchid materials. WCT participated in the design and coordination, and drafted the manuscript. HHC initiated the project, contributed to the experimental design and edited the manuscript. All authors read and approved the final manuscript.

## Supplementary Material

Additional file 1 **Length distribution of assembled contigs and singletons**. This table summarizes the number of contigs and singletons in different length distribution. Click here for file

Additional file 2 **Summary of component reads per assembly**. This table summarizes the number of component reads assembled into contigs. Click here for file

Additional file 3 **Gene Families identified by BLAST annotation of *Phalaenopsis *transcriptome**. This table summarizes the BLAST results of all Unigenes against *Arabidopsis *proteome and then categorized by *Arabidopsis *gene families. Click here for file

Additional file 4 **Expressed sequence tags with substantial similarity to terpenoid backbone biosynthetic genes**. This table summarizes the number of unigenes and reads in each step of terpenoid biosynthetic pathway. Click here for file
