# Supplementary material for: Gene discovery using next-generation pyrosequencing to develop ESTs for Phalaenopsis orchids
Source: BMC Genomics. 2011 Jul 12;12:360. doi: 10.1186/1471-2164-12-360 (PMC3146457; doi:10.1186/1471-2164-12-360)
Supplement: Additional file 1 — Length distribution of assembled contigs and singletons. This table summarizes the number of contigs and singletons in different length distribution. [file 1471-2164-12-360-S1.DOC]

Table S1. Length distribution of assembled contigs and singletons

| **Nucleotides length (bp)** | **Contigs** | **Singletons** |
| --- | --- | --- |
| 0-49 | 0 | 150 |
| 50-99 | 24 | 4,730 |
| 100-199 | 466 | 7,410 |
| 200-299 | 4,422 | 22,344 |
| 300-399 | 1,177 | 146 |
| 400-499 | 791 | 1 |
| 500-599 | 458 | 0 |
| 600-699 | 321 | 0 |
| 700-799 | 194 | 0 |
| 800-899 | 109 | 0 |
| 900-999 | 81 | 0 |
| 1,000-1,499 | 147 | 0 |
| 1,500-1,999 | 36 | 0 |
| >2,000 | 8 | 0 |
| **Total** | 8,233 | 34,780 |
| **Maximum length** | 4,234 bp | 416 bp |
| **Average length** | 364 bp | 201 bp |
