# Supplementary material for: Gene discovery using next-generation pyrosequencing to develop ESTs for Phalaenopsis orchids
Source: BMC Genomics. 2011 Jul 12;12:360. doi: 10.1186/1471-2164-12-360 (PMC3146457; doi:10.1186/1471-2164-12-360)
Supplement: Additional file 2 — Summary of component reads per assembly. This table summarizes the number of component reads assembled into contigs. [file 1471-2164-12-360-S2.DOC]

Table S2. Summary of component reads per assembly.

| **Number of reads** | **Number of contigs** |
| --- | --- |
| 2 to 10 | 6,441 |
| 11 to 20 | 1,021 |
| 21-30 | 307 |
| 31-40 | 143 |
| 41-50 | 92 |
| 51-100 | 130 |
| 101-150 | 32 |
| 151-200 | 12 |
| > 200 | 55 |
