# Supplementary material for: Gene discovery using next-generation pyrosequencing to develop ESTs for Phalaenopsis orchids
Source: BMC Genomics. 2011 Jul 12;12:360. doi: 10.1186/1471-2164-12-360 (PMC3146457; doi:10.1186/1471-2164-12-360)
Supplement: Additional file 3 — Gene Families identified by BLAST annotation of Phalaenopsis transcriptome. This table summarizes the BLAST results of all Unigenes against Arabidopsis proteome and then categorized by Arabidopsis gene families. [file 1471-2164-12-360-S3.DOC]

Table S3. Gene Families identified by BLAST annotation of *Phalaenopsis* transcriptome

| Gene Family | Number of  Unigenes | Percentage of Unigenes | Gene Family | Number of  Unigenes | Percentage of Unigenes |
| --- | --- | --- | --- | --- | --- |
| 14-3-3 proteins | 14 | 0.29% | HRT Transcription Factor Family | 1 | 0.02% |
| 14-3-3s | 9 | 0.19% | HSF Transcription Factor Family | 9 | 0.19% |
| AAAP family | 21 | 0.43% | HSP70s | 16 | 0.33% |
| ABC Superfamily | 43 | 0.89% | Hypothetical proteins | 2 | 0.04% |
| ABC transporters | 90 | 1.86% | IDZ Gene Family | 4 | 0.08% |
| ABC transporters (Smart Lab) | 58 | 1.20% | Inorganic Solute Cotransporters | 76 | 1.57% |
| ABI3VP1 Transcription Factor Family | 3 | 0.06% | Ion Channel Families | 55 | 1.14% |
| ACBP60s | 7 | 0.14% | IQD Protein Family | 9 | 0.19% |
| Acyl Lipid Metabolism Family | 524 | 10.84% | JUMONJI Transcription Factor Family | 3 | 0.06% |
| AGC Family | 36 | 0.74% | Kinesins | 35 | 0.72% |
| Aldehyde Dehydrogenase Superfamily | 40 | 0.83% | Lateral Organ Boundaries Gene Family | 6 | 0.12% |
| Alfin-like Transcription Factor Family | 9 | 0.19% | Leucine-rich repeat extensin | 6 | 0.12% |
| Antiporter Superfamily | 3 | 0.06% | Lipid Metabolism Gene Families | 101 | 2.09% |
| Antiporters | 44 | 0.91% | MADS-box Transcription Factor Family | 18 | 0.37% |
| AP2-EREBP Transcription Factor Family | 27 | 0.56% | Magnesium Transporter Gene Family | 14 | 0.29% |
| ARF Transcription Factor Family | 20 | 0.41% | MAP Kinase (MAPK) Family | 16 | 0.33% |
| ARIADNE gene family | 3 | 0.06% | MAP Kinase Kinase (MAPKK) Family | 9 | 0.19% |
| ARID Transcription Factor Family | 7 | 0.14% | MAP Kinase Kinase Kinase (MAPKKK) Family | 58 | 1.20% |
| BBR/BPC-family of GAGA-motif binding transcription factors | 3 | 0.06% | MAP Kinase Kinase Kinase Kinase (MAPKKKK) Family | 1 | 0.02% |
| bHLH Transcription Factor Family | 37 | 0.77% | Mechanosensitive Ion Channel Family | 11 | 0.23% |
| bZIP Transcription Factor Family | 22 | 0.46% | MIP family | 24 | 0.50% |
| BZR Transcription Factor Family | 6 | 0.12% | Miscellaneous Membrane Protein Families | 364 | 7.53% |
| C2C2-CO-like Transcription Factor Family | 13 | 0.27% | MLO proteins | 6 | 0.12% |
| C2C2-Gata Transcription Factor Family | 6 | 0.12% | Monolignol Biosynthesis | 92 | 1.90% |
| C2C2-YABBY Transcription Factor Family | 5 | 0.10% | MYB Transcription Factor Family | 17 | 0.35% |
| C2H2 Transcription Factor Family | 74 | 1.53% | MYB-Related Transcription Factor Family | 6 | 0.12% |
| C3H Transcription Factor Family | 61 | 1.26% | Myosin | 9 | 0.19% |
| Carbohydrate Esterase Gene Families | 41 | 0.85% | Nodulin-like protein family | 31 | 0.64% |
| CBL-interacting serione-threonine Protein Kinases (AtCIPKs) | 37 | 0.77% | Organic Solute Cotransporters | 243 | 5.03% |
| CCAAT-HAP2 Transcription Factor Family | 1 | 0.02% | Orphan Transcription Factor Family | 2 | 0.04% |
| CCAAT-HAP3 Transcription Factor Family | 3 | 0.06% | PCBPs | 11 | 0.23% |
| CCAAT-HAP5 Transcription Factor Family | 11 | 0.23% | PHD Gene Family | 2 | 0.04% |
| CDPKs | 33 | 0.68% | PHD Transcription Factor Family | 8 | 0.17% |
| Chloroplast and Mitochondria gene families | 53 | 1.10% | Phospholipase D (Zarsky group) | 18 | 0.37% |
| Class III peroxidase | 17 | 0.35% | Phosphoribosyltransferases (PRT) | 9 | 0.19% |
| COBRA Gene Family | 7 | 0.14% | Plant Cell Wall Biosynthesis Families | 57 | 1.18% |
| Core Cell Cycle Genes | 29 | 0.60% | Plant U-box protein (PUB) | 56 | 1.16% |
| CPP Transcription Factor Family | 2 | 0.04% | Polysaccharide Lyase Gene Families | 19 | 0.39% |
| CRKs | 4 | 0.08% | PP2C-type phosphatases | 93 | 1.92% |
| Cytochrome b5 | 6 | 0.12% | PPI | 1 | 0.02% |
| Cytochrome P450 | 94 | 1.94% | Primary Pumps (ATPases) Gene Families | 113 | 2.34% |
| Cytoplasmic ribosomal protein gene family | 210 | 4.35% | Primary Pumps (ATPases) Gene Family (2) | 45 | 0.93% |
| Cytoskeleton | 7 | 0.14% | Protein tyrosine phosphatase (PTP) family | 20 | 0.41% |
| Dof family | 8 | 0.17% | Rad5/16-like gene family | 3 | 0.06% |
| E2F-DP Transcription Factor Family | 3 | 0.06% | RAV Transcription Factor Family | 6 | 0.12% |
| EF-hand containing proteins | 151 | 3.12% | RCI2 gene Family | 3 | 0.06% |
| EICBPs | 13 | 0.27% | Receptor kinase-like protein family | 140 | 2.90% |
| EIL Transcription Factor Family | 4 | 0.08% | Response Regulator | 12 | 0.25% |
| Eukaryotic Initiation Factor Gene Family | 142 | 2.94% | SBP Transcription Factor Family | 10 | 0.21% |
| EXO70 exocyst subunit family | 12 | 0.25% | Short Under Blue Light (SUBs) | 7 | 0.14% |
| Expansins | 11 | 0.23% | Single gene-encoded CBPs | 21 | 0.43% |
| F-Box Proteins | 2 | 0.04% | Single Myb Histone (SMH) | 1 | 0.02% |
| FH2 proteins | 8 | 0.17% | Strubbelig Receptor Family | 11 | 0.23% |
| FtsH: AAA ATP-dependent zinc metallopeptidase | 17 | 0.35% | Structural Maintenance of Chromosomes family | 5 | 0.10% |
| G2-like Transcription Factor Family | 15 | 0.31% | Subtilisin-like Serine Proteases | 50 | 1.03% |
| GAD | 2 | 0.04% | Sucrose-H+ symporters | 7 | 0.14% |
| GeBP Transcription Factor Family | 5 | 0.10% | Sulfurtransferasese / Rhodanese Family | 12 | 0.25% |
| Glutathione S-transferase Family | 37 | 0.77% | TCP transcription factor family | 5 | 0.10% |
| Glycoside Hydrolase Gene Families | 257 | 5.32% | TGA3-like | 2 | 0.04% |
| Glycosyltransferase Gene Families | 204 | 4.22% | tify family | 4 | 0.08% |
| GRAS Transcription Factor Family | 35 | 0.72% | Trehalose Biosynthesis Gene Families | 31 | 0.64% |
| GRF Transcription Factor Family | 3 | 0.06% | Trihelix Transcription Factor Family | 13 | 0.27% |
| Histidine Kinase | 16 | 0.33% | TUB Transcription Factor Family | 17 | 0.35% |
| Histidine Phosphotransfer Proteins | 6 | 0.12% | WRKY Transcription Factor Superfamily | 19 | 0.39% |
| Homeobox Transcription Factor Family | 60 | 1.24% | zinc finger-homeobox gene family | 7 | 0.14% |
