# Supplementary material for: Gene discovery using next-generation pyrosequencing to develop ESTs for Phalaenopsis orchids
Source: BMC Genomics. 2011 Jul 12;12:360. doi: 10.1186/1471-2164-12-360 (PMC3146457; doi:10.1186/1471-2164-12-360)
Supplement: Additional file 4 — Expressed sequence tags with substantial similarity to terpenoid backbone biosynthetic genes. This table summarizes the number of unigenes and reads in each step of terpenoid biosynthetic pathway. [file 1471-2164-12-360-S4.DOC]

**Table S4.** Expressed sequence tags with substantial similarity to terpenoid backbone biosynthetic genes

| EST designation | EC Number | Length (bp) | Description | Species | E-value | Number of ESTs |
| --- | --- | --- | --- | --- | --- | --- |
| Contig 00396 | 2.5.1.29 | 410 | GGDP synthase | *Tagetes erecta* | 3E-21 | 6 |
| Contig 00468 | 2.5.1.10 | 406 | farnesyl pyrophosphare synthase | *Musa acuminata* | 3E-18 | 11 |
| Contig 01180 | 2.5.1.1 | 396 | geranyl diphosphate synthase large subunit | *Antirrhinum majus* | 2E-35 | 10 |
| Contig 02737 | 1.17.7.1 | 321 | predicted protein | *Populus trichocarpa* | 7E-51 | 6 |
| Contig 02761 | 2.2.1.7 | 210 | predicted protein | *Populus trichocarpa* | 2E-11 | 13 |
| Contig 03302 | 1.17.7.1 | 982 | 4-hydroxy-3-methylbut-2-en-1-yl diphosphate synthase | *Hevea brasiliensis* | 1E-47 | 24 |
| Contig 03636 | 2.5.1.10 | 583 | farnesyl pyrophosphate synthase | *Chimonanthus praecox* | 9E-75 | 6 |
| Contig 03668 | 1.1.1.34 | 266 | 3-hydroxy-3-methylglutaryl-coenzyme A reductase 3 | *Oryza sativa* | 1E-11 | 4 |
| Contig 04655 | 1.17.1.2 | 1274 | hydroxymethylbutenyl diphosphate reductase | *Oncidium hybrid cultivar* | 0 | 32 |
| Contig 04715 | 2.3.3.10 | 434 | hydroxymethylglutaryl-CoA synthase, putative | *Ricinus communis* | 3E-73 | 7 |
| Contig 05410 | 2.2.1.7 | 563 | 1-deoxyxylulose-5-phosphate synthase, putative | *Ricinus communis* | 2E-97 | 9 |
| Contig 06260 | 2.3.3.10 | 654 | hydroxymethylglutaryl-CoA synthase | *Oryza brachyantha* | 4E-58 | 9 |
| Contig 06334 | 1.3.1.83 | 280 | geranyl-geranyl reductase | *Elaeis oleifera* | 3E-24 | 5 |
| Contig 06765 | 2.5.1.85 | 498 | solanesyl diphosphate synthase | *Hevea brasiliensis* | 1E-76 | 10 |
| Contig 06936 | 1.1.1.34 | 262 | hmg-CoA reductase, putative | *Ricinus communis* | 2E-39 | 4 |
| Contig 07023 | 2.2.1.7 | 351 | 1-deoxy-D-xylulose-5-phosphate synthase | *Oryza sativa* | 5E-57 | 5 |
| Contig 07058 | 2.2.1.7 | 618 | 1-deoxy-D-xylulose 5-phosphate synthase | *Pueraria montana var. lobata* | 5E-98 | 10 |
| Contig 07119 | 2.5.1.29 | 314 | putative geranylgeranyl pyrophosphate synthase 1 | *Zea mays* | 3E-24 | 5 |
| Contig 07274 | 2.2.1.7 | 389 | predicted protein | *Populus trichocarpa* | 1E-62 | 7 |
| Contig 07353 | 2.2.1.7 | 363 | chloroplast 1-deoxy-D-xylulose-5-phosphate synthase | *Elaeis guineensis* | 2E-52 | 9 |
| Contig 07465 | 2.2.1.7 | 363 | putative 1-deoxy-D-xylulose 5-phosphate synthase | *Hevea brasiliensis* | 1E-48 | 7 |
| Contig 07590 | 1.1.1.34 | 264 | 3-hydroxy-3-methylglutaryl coenzyme A reductase 5 | *Medicago truncatula* | 8E-40 | 12 |
| Contig 07786 | 5.3.3.2 | 543 | isopentenyl diphosphate isomerase 2 | *Nicotiana tabacum* | 4E-86 | 14 |
| Contig 07972 | 2.5.1.- | 445 | predicted protein | *Populus trichocarpa* | 1E-33 | 5 |
| FXQQ4OB01A54PC | 2.7.1.148 | 253 | predicted protein | *Populus trichocarpa* | 1E-24 | 1 |
| FXQQ4OB01A5FRM | 1.1.1.267 | 217 | 1-deoxy-D-xylulose 5-phosphate reductoisomerase | *Vanda hybrid cultivar* | 4E-30 | 1 |
| FXQQ4OB01A77YH | 2.7.1.36 | 225 | mevalonate kinase | *Hevea brasiliensis* | 3E-10 | 1 |
| FXQQ4OB01A8UWV | 4.1.1.33 | 236 | Os02g0109100 | *Oryza sativa* | 2E-18 | 1 |
| FXQQ4OB01ANEGU | 2.3.3.10 | 263 | HMG-CoA synthase | *Nicotiana langsdorffii x Nicotiana sanderae* | 6E-23 | 1 |
| FXQQ4OB01AQHEZ | 2.7.4.2 | 207 | phosphomevalonate kinase | *Zea mays* | 2E-13 | 1 |
| FXQQ4OB01B5QFB | 2.3.1.9 | 240 | predicted protein | *Populus trichocarpa* | 1E-33 | 1 |
| FXQQ4OB01C52HX | 2.7.4.2 | 240 | phosphomevalonate kinase | *Zea mays* | 4E-19 | 1 |
| FXQQ4OB01C7A2K | 4.6.1.12 | 256 | unnamed protein product | *Vitis vinifera* | 5E-18 | 1 |
| FXQQ4OB01C8YH1 | 2.7.4.2 | 237 | Os03g0253100 | *Oryza sativa* | 6E-21 | 1 |
| FXQQ4OB01CHNJK | 1.1.1.267 | 243 | 1-deoxy-D-xylulose 5-phosphate reductoisomerase | *Vanda hybrid cultivar* | 2E-34 | 1 |
| FXQQ4OB01CQGWD | 2.3.1.9 | 204 | predicted protein | *Populus trichocarpa* | 4E-27 | 1 |
| FXQQ4OB01DA11A | 2.3.1.9 | 204 | predicted protein | *Populus trichocarpa* | 4E-27 | 1 |
| FXQQ4OB01DTHJB | 2.7.1.148 | 239 | unnamed protein product | *Vitis vinifera* | 5E-24 | 1 |
| FXQQ4OB01DZ4ZM | 2.7.1.36 | 262 | predicted protein | *Populus trichocarpa* | 2E-12 | 1 |
| FXQQ4OB01EKHLI | 2.2.1.7 | 228 | 1-deoxy-D-xylulose-5-phosphate synthase | *Bixa orellana* | 3E-23 | 1 |
| FXQQ4OB01ELHAO | 1.3.1.83 | 245 | geranylgeranyl hydrogenase | *Triticum aestivum* | 7E-33 | 1 |
| FXQQ4OB02F2B4B | 4.1.1.33 | 240 | diphosphomevalonate decarboxylase-like protein | *Arabidopsis thaliana* | 1E-26 | 1 |
| FXQQ4OB02F34MU | 1.1.1.267 | 223 | 1-deoxy-D-xylulose 5-phosphate reductoisomerase | *Vanda hybrid cultivar* | 2E-23 | 1 |
| FXQQ4OB02F5RI4 | 2.2.1.7 | 120 | 1-deoxyxylulose-5-phosphate synthase, putative | *Ricinus communis* | 2E-13 | 1 |
| FXQQ4OB02FSOIX | 1.1.1.267 | 234 | 1-deoxy-D-xylulose 5-phosphate reductoisomerase | *Vanda hybrid cultivar* | 1E-33 | 1 |
| FXQQ4OB02FZRPD | 4.1.1.33 | 229 | Os02g0109100 | *Oryza sativa* | 2E-31 | 1 |
| FXQQ4OB02G79E1 | 1.3.1.83 | 246 | predicted protein | *Physcomitrella patens* subsp*. patens* | 3E-36 | 1 |
| FXQQ4OB02GHMH7 | 1.3.1.83 | 254 | geranylgeranyl reductase | *Olea europaea* | 5E-23 | 1 |
| FXQQ4OB02GREV7 | 2.7.4.2 | 237 | phosphomevalonate kinase | *Zea mays* | 5E-20 | 1 |
| FXQQ4OB02GTT6C | 1.1.1.267 | 261 | 1-deoxy-D-xylulose 5-phosphate reductoisomerase | *Vanda hybrid cultivar* | 2E-24 | 1 |
| FXQQ4OB02GYPHN | 1.1.1.267 | 235 | 1-deoxy-D-xylulose 5-phosphate reductoisomerase | *Vanda hybrid cultivar* | 2E-33 | 1 |
| FXQQ4OB02HV4IR | 4.6.1.12 | 247 | 2-C-methyl-D-erythritol 2,4-cyclodiphosphate synthase | *Nicotiana langsdorffii x Nicotiana sanderae* | 2E-18 | 1 |
| FXQQ4OB02I0EMJ | 2.3.1.9 | 239 | predicted protein | *Populus trichocarpa* | 5E-34 | 1 |
| FXQQ4OB02I6T82 | 2.7.4.2 | 234 | Os03g0253100 | *Oryza sativa* | 6E-16 | 1 |
| FXQQ4OB02IMKP1 | 1.3.1.83 | 225 | putative geranylgeranyl reductase | *Silene viscosa* | 1E-34 | 1 |
| FXQQ4OB02IPLCS | 2.7.1.148 | 236 | unnamed protein product | *Vitis vinifera* | 2E-29 | 1 |
| FXQQ4OB02IT1C7 | 2.7.1.148 | 236 | unnamed protein product | *Vitis vinifera* | 2E-29 | 1 |
